# eIF3d and eIF4G2 mediate an alternative mechanism of cap-dependent but eIF4E-independent translation initiation

**DOI:** 10.1016/j.jbc.2025.108317

**Published:** 2025-02-17

**Authors:** Jacob N.K. Quartey, Dixie J. Goss

**Affiliations:** 1 Ph.D. Program in Biochemistry, The Graduate Center of the City University of New York, New York, New York, USA; 2 Department of Chemistry, Hunter College of the City University of New York, New York, New York, USA; 3 Ph.D. Program in Chemistry, The Graduate Center of the City University of New York, New York, New York, USA

**Keywords:** 7-methylguanosine cap (m^7^G) cap, fluorescence anisotropy, hypoxia, eIF3d, eIF4G2

## Abstract

Initiation of translation for the majority of eukaryotic mRNAs is mediated by a 5′ cap structure to which the eukaryotic initiation factor 4E (eIF4E) binds. Inhibition of the activity of eIF4E by 4EBP-1 does not prevent the translation of a number of cellular capped mRNAs, indicative of the existence of previously unexplored mechanisms for the translation of these capped mRNAs without the requirement of eIF4E. eIF4G2, also known as death-associated protein 5 (DAP5), a homolog of eIFGI that lacks the eIF4E binding domain, utilizes eIF3d (a subunit of eIF3) to promote the translation of a subset of these mRNAs. Using fluorescence anisotropy-based equilibrium binding studies, we provide the first quantitative evidence of the recruitment of eIF3d as well as eIF3d and eIFG2 complexes to a subset of human mRNAs. Our quantitative studies demonstrate the critical role a fully methylated 5′ mRNA cap structure plays in the recognition and recruitment of eIF3d, as well as the eIF3d and eIFG2 complex. By using luciferase reporter-based *in vitro* translation assays, we further show that cap-recognition ability correlates with the efficiency of translation of these mRNAs. Essentially, by preferably utilizing eIF3d and eIFG2, specific mRNA subsets are still able to translate in a cap-dependent manner even when eIF4E is sequestered. Our findings offer new insight into the use of eIF3d and eIF4G2 as an alternative for growth and survival under conditions of cellular stress. This novel mechanism of translation may offer new targets for therapeutic regulation of mRNA translation.

Protein synthesis is a tightly regulated process which is made possible with the help of the powerful synthetic machinery; the ribosomes and a host of initiation factors in the cell (1, 2). This complex system coordinates several distinct steps which include initiation, elongation, termination, folding, and quality control of the resulting product (3, 4). Translation initiation in eukaryotes begins with the recognition of the 7-methylguanosine (m^7^G) cap structure by eukaryotic initiation factor 4E (eIF4E) (5, 6, 7). The binding of the m^7^G cap structure by eIF4E subsequently leads to the recruitment of eIF4GI and eIF4A which in turn recruit the 43S preinitiation complex, consisting of the 40S ribosome loaded with methionyl-tRNA and additional eukaryotic initiation factors to form the 48S initiation complex (8, 9). The 48S initiation complex then scans the mRNA from the 5′end to locate the start codon (1, 5, 10). Upon recognition of the start codon, the 60S ribosomal subunit then joins with the 40S subunit to assemble an actively translating 80S ribosome (1, 5). This mechanism of translation occurs so long as the cell is operating under normal conditions such as availability of nutrients, normoxia, and homeostasis (1, 2, 5).

Under conditions of cellular stress such as nutrient limitation, hypoxia, viral infection, and proteotoxic stress, canonical cap-dependent translation is inhibited (11, 12). Human 4E binding protein 1 (4EBP-1), a negative regulator of eIF4E activity, is hypophosphorylated, making it active thus binding efficiently to eIF4E and blocking translation (13, 14). As a result of eIF4GI and hypophosphorylated 4EBP-1 sharing the same binding site on eIF4E, active 4EBP-1 blocks the eIF4GI binding site on eIF4E thereby inhibiting eIF4F formation and subsequently translation (15, 16). Although 4EBP-1 sequestration of eIF4E inhibits translation to a large extent for a majority of mRNAs in the cell, a number of capped mRNAs are still able to translate (17). The mechanisms by which these mRNAs are able to translate despite sequestration of eIF4E have generated a lot of interest recently. For example, during epithelial-mesenchymal transition (EMT) and metastasis, there is significant stress in the cell, and as a result of mammalian target of rapamycin complex 1 (mTORC1) inhibition, the canonical method of translation initiation which involves the recognition and binding of eIF4E to the 5′ mRNA cap structure, is impaired (17, 18, 19, 20). Despite the harsh cellular conditions, these mRNAs must still be able to translate in order for these cells to function properly. However, during metastasis and EMT, the majority of mRNA translation must be cap-dependent despite the inhibition of mTORC1 and eIF4E activity (17, 20). This leads to the controversial question as to how mRNAs that encode essential biological functions for metastasis, cancer cell proliferation, and EMT are translated during stress conditions in the cell. Indeed, there must be other mechanisms of cap-dependent mRNA translation essential for cell survival during stress which have previously not been fully explored.

Recent studies have shown that eIF3d, a subunit of the large 800 kDa eIF3 multisubunit protein complex, is able to mediate specialized mRNA translation under conditions of mTORC1 inhibition (17, 18, 21, 22). By virtue of the cap-binding property of eIF3d, it is able to drive alternate mechanisms of selective cap-dependent mRNA translation in the event of eIF4E sequestration (23, 24). Human eIF3d consists of 548 amino acid residues and has a molecular weight of about 66 kDa (25). It possesses a unique “RNA gate” which lies between residues 285 and 299 (22, 25) (Fig. 1, *A* and *C*). eIF3d is highly conserved among different species including *Drosophila*, with data from sequence tag databases indicating that it is ubiquitously and abundantly expressed (26, 27). eIF3d is also overexpressed in many types of cancers such as cervical, ovarian, lung, and renal cell carcinomas, and may serve as a potential novel biomarker of disease progression (28, 29, 30, 31, 32). For example, the overexpression of eIF3d in lung adenocarcinoma has been shown to be a new independent marker of poor survival in patients (31).

**Figure 1:**
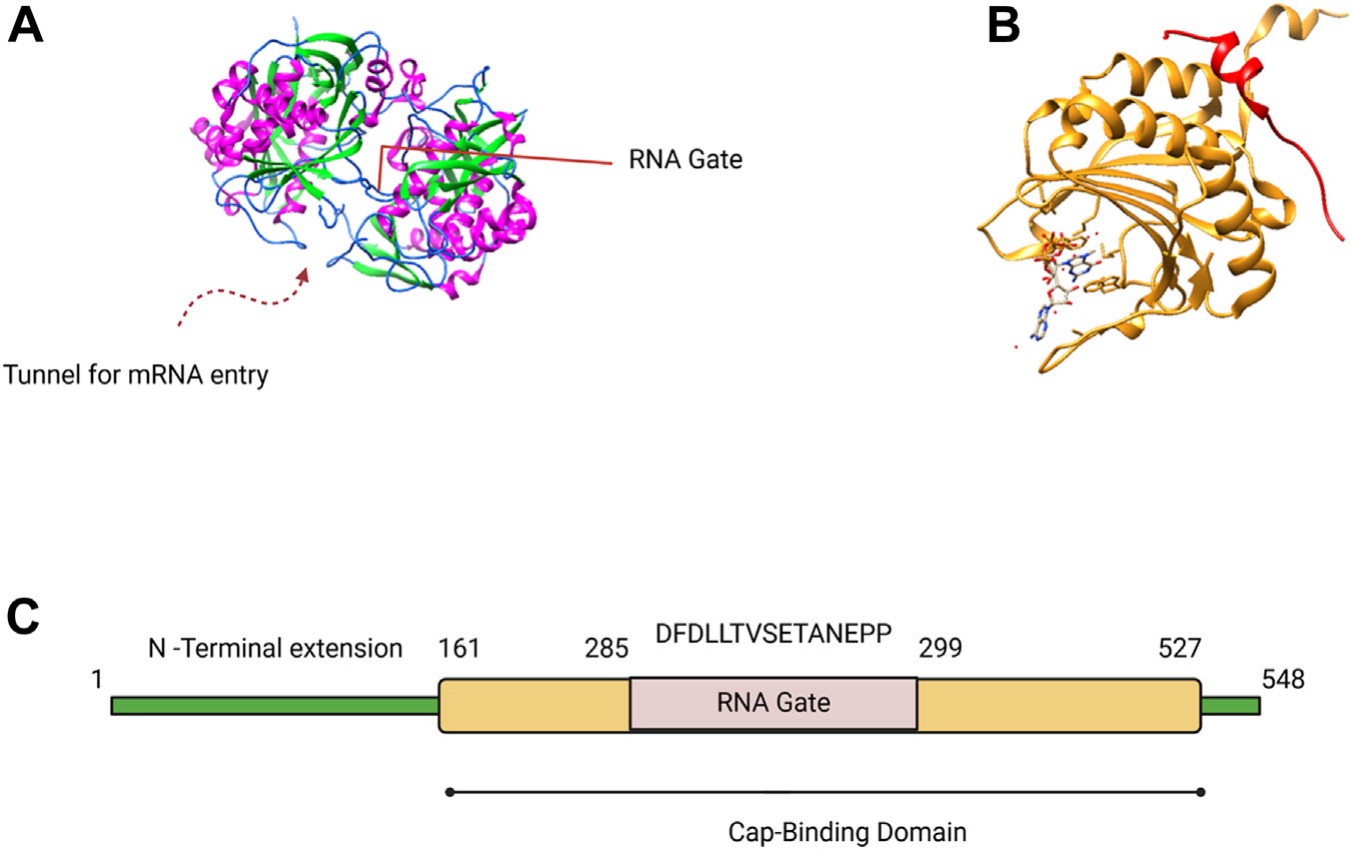
**Structure of eIF3d and eIF4E cap binding domains.***A*, the cap binding domain of eIF3d showing the tunnel for mRNA entry and RNA gate, which are unique to eIF3d but absent in the cap-binding pocket of eIF4E. α helices are colored in *purple* and β strands in *green*. Figure was prepared with data in PDB ID code 5K4B using UCSF Chimera software (https://www.cgl.ucsf.edu/chimerax/). *B*, cap binding pocket of eIF4E, shown in *gold*, in contact with the m^7^G cap which is represented with a ball and stick model. 4EBP-1 (amino acid residues 36–70) shown in *red* is bound to eIF4E. Figure was prepared with data in PDB ID code 1WKW using UCSF Chimera software. *C*, schematic showing the domain architecture of human eIF3d. Human eIF3d consists of an unstructured N-terminal region, as well as a cap binding domain, found between amino acid residues 161 and 527. Within the cap-binding domain is the RNA gate which spans amino acid residues 285 to 299. The amino acid residues that make up the RNA gate are shown above the domain box. 4EBP-1, 4E binding protein 1; eIF, eukaryotic initiation factor; m^7^G, 7-methylguanosine; PDB, Protein Data Bank.

eIF4G2 also known as death-associated protein 5 (DAP5), has also been shown to have increased expression levels under conditions of cellular stress (33, 34). eIF4G2 is a member of the eIF4G protein family that lacks the poly A binding protein (PABP) and eIF4E interaction sites which are present on eIF4GI (20, 35, 36) (Fig. 2). eIF4G2 shares 65% homology with eIF4GI and is expressed abundantly in proliferating cells (37). The MIF4G domain of eIF4G2 plays a vital role in its interaction with eIF3 and eIF4A (36). Under conditions of cell stress in cancer cells for example, eIF4G2 is able to drive the cap-independent translation of particular mRNA subsets (20, 36, 37, 38). These mRNA subsets typically have strong secondary structural elements in their 5′UTRs to which eIF4G2 is recruited (38, 39). eIF4G2 has recently been shown to bind directly to the 5′UTRs of hypoxia inducible factor-1 alpha (HIF-1α) , fibroblast growth factor-9 (FGF-9), and p53 encoding mRNAs, and promote their translation in a cap-independent manner (38). The internal ribosome entry site driven translation of apoptotic peptidase activating factor 1 (APAF1) , Bcl2-associated athanogene 1(BAG1), and the MYC oncogene mRNAs are also promoted by eIF4G2 (20, 33, 40).

**Figure 2:**
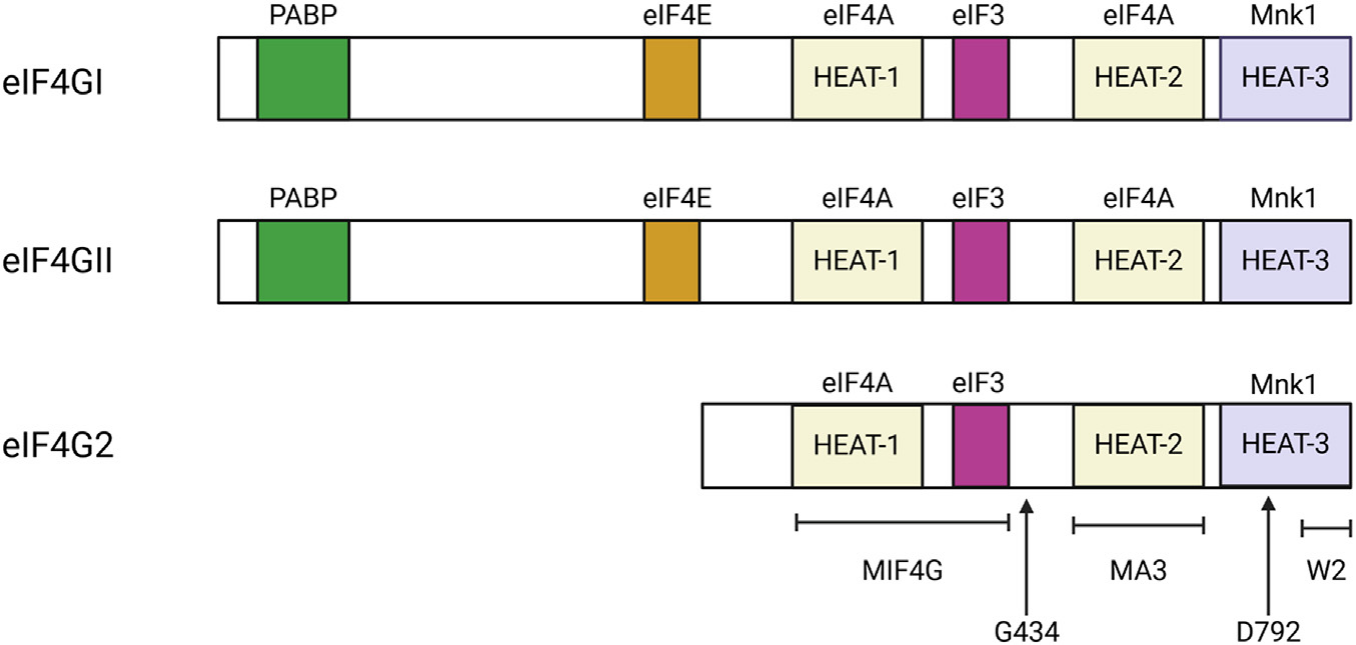
**Domain architecture of the human eIF4G protein family.** eIF4G2 lacks the poly(A) binding protein (PABP) and eIF4E domains which can be found on eIF4GI and eIF4GII. The *colored boxes* show the domains of interaction for each eIF4G protein family member with the corresponding factor specified above the domain boxes. Each eIF4G protein family member has three HEAT domains, namely HEAT-1, HEAT-2, and HEAT-3, respectively. The MIF4G domain of eIF4G2 is crucial in mediating its interaction with eIF4A and eIF3. The cleavage sites on eIF4G2 are shown with *arrows*. G434 is the viral protease 2A cleavage site, whereas D792 is the caspase cleavage site. eIF4E, eukaryotic initiation factor 4E.

Emerging studies now reveal a whole new interesting dimension to our understanding of eIF4G2 function during the cellular stress response. These studies show that eIF4G2 is able to participate in the cap-dependent translation of certain mRNAs during stress. This unique mode of translation initiation is contingent upon the strong association between eIF3d and eIF4G2. Indeed, recent mass spectrometric data reveal eIF4G2 as a very strong binding partner of eIF3d (18).

By interacting with each other, eIF3d and eIF4G2 are able to mediate a previously unexplored form of cap-dependent translation initiation (17, 18, 41). This alternative form of translation initiation ensures that cells are still able to survive, even under conditions where eIF4E is sequestered (17, 41). This eIF3d and eIF4G2 mediated form of translation is necessary for EMT, invasion, cell migration, and metastasis (41). In this report, we provide the first quantitative and mechanistic insight into the utilization of eIF3d (the d-subunit of eIF3) as an alternative for the translation of specific mRNA subsets under conditions where mTORC1 activity is impaired. We show that eIF4G2 is crucial in mediating the activities of eIF3d under such conditions. In essence, particular mRNA subsets are able to be translated *via* an alternate method that utilizes eIF3d and eIF4G2. This form of translation proceeds *via* a cap-dependent manner and does not require eIF4E.

## Results

### eIF3d binds directly to m^7^GpppA capped mRNA

**Figure 3:**
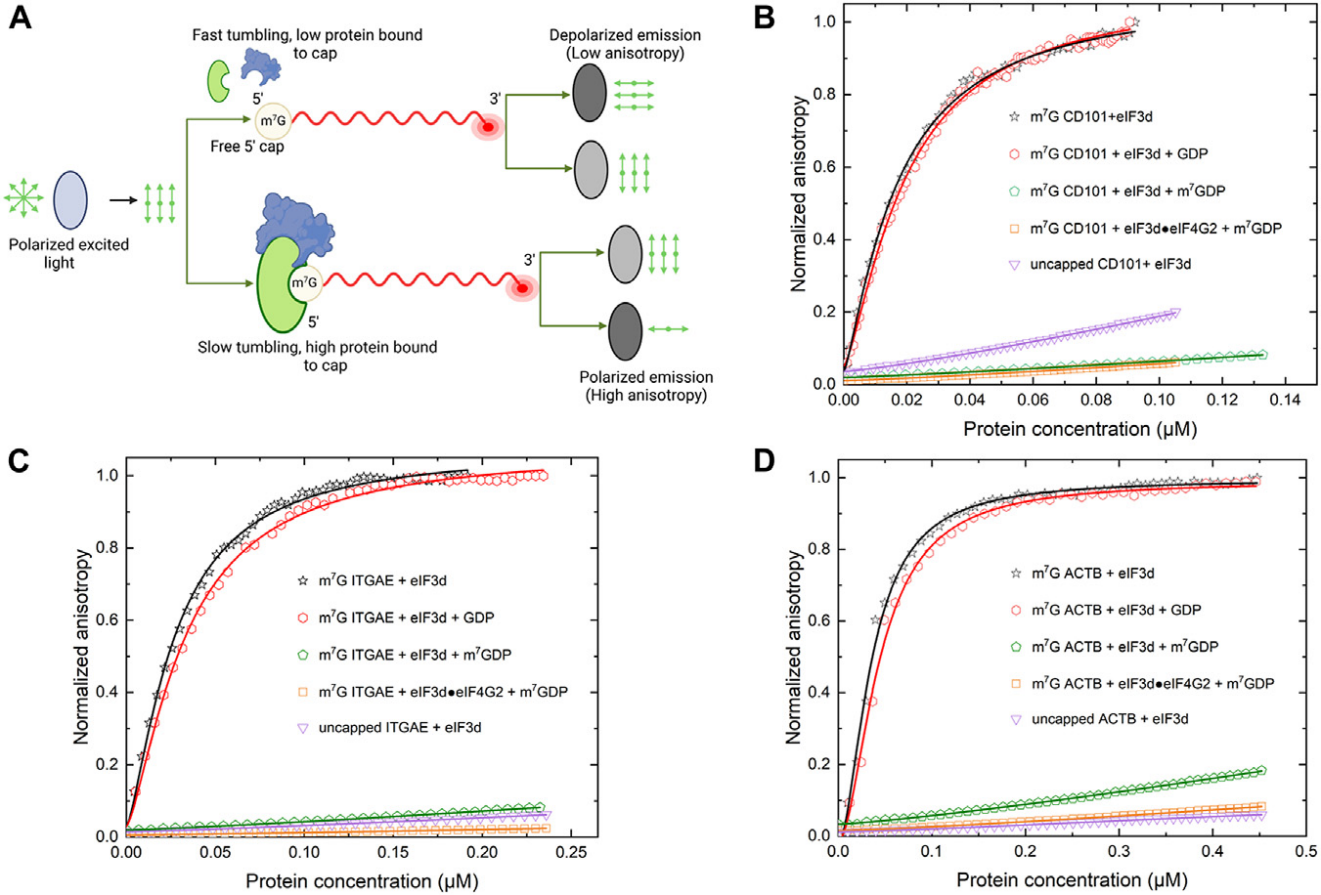
**Equilibrium-binding titrations of fluorescein-labeled m**^**7**^**G capped 5′UTRs with eIF3d.***A*, cartoon showing the fluorescence anisotropy-based equilibrium binding assay. Equilibrium-binding titrations of fluorescein-labeled m^7^G capped 5′UTRs of mRNAs coding for (*B*), CD101, (*C*), ITGAE and (*D*), ACTB with either eIF3d alone, eIF3d in the presence of GDP, eIF3d in the presence of m^7^GDP, or a complex of eIF3d and eIF4G2 in the presence of m^7^GDP. The normalized anisotropy change for the interaction between the fluorescein-labeled, but uncapped, 5′ UTRs with eIF3d was included as a control. Briefly, 10 nM of fluorescein-labeled capped or uncapped mRNAs were titrated with increasing concentration of protein or protein/protein complex in the titration buffer at 25 °C. The anisotropy at each titration point was measured using excitation and emission wavelengths of 495 nm and 520 nm, respectively. Data points correspond to an average of three independent anisotropy measurements. The curves represent the nonlinear fits that were used to obtain the averages and standard deviations for the corresponding K_d_ values presented in Table 1. ACTB, β-actin; CD101, cluster of differentiation 101; eIF, eukaryotic initiation factor; ITGAE, integrin subunit alpha E; m^7^GDP, 7-methylguanosine diphosphate.

Recently, the immunoglobulin superfamily member 2 (IGSF2) also known as cluster of differentiation 101 (CD101), and integrin subunit alpha E (ITGAE), also known as CD103 (cluster of differentiation 103), have been identified as being able to utilize the noncanonical eIF3d pathway in translation initiation (18). These identifications were made using genome-wide translatomic and ingenuity pathway analysis (18). β-actin (ACTB) mRNA on the other hand, is well-known to utilize the canonical eIF4E cap-dependent translation mechanism (7). To investigate the binding affinities of eIF3d to the mRNA transcripts, fluorescence anisotropy-based equilibrium assays were performed. In this assay, binding curves are produced as a result of anisotropy changes that result from titrated proteins binding to RNA molecules which are covalently labeled with fluorescein at the 3′end (Fig. 3*A*). The binding affinities of eIF3d to the 5′UTRs of mRNAs coding for CD101, ITGAE, and ACTB that were either m^7^G capped, or uncapped, were determined.

Our binding data indicate that eIF3d interacts with the m^7^G capped transcripts much stronger than it does with the uncapped mRNA transcripts (Fig. 3 and Table 1). The measured equilibrium dissociation constants (K _d_'s) for eIF3d binding to 5′ m^7^G capped CD101, ITGAE, and ACTB were (17 ± 2) nM, (26 ± 3) nM, and (35 ± 4) nM, respectively (Table 1). eIF3d did not show any appreciable binding to the uncapped transcripts tested (Fig. 3). This observation suggests that the m^7^G cap plays a crucial role in the recruitment of eIF3d. Previous bioinformatics and sequence database analysis showed that the 5′UTRs of mRNAs coding for CD101, ITGAE, and ACTB did not possess significant secondary or structural motifs (18). Thus the equilibrium dissociation constants measured may solely be a result of the interaction between eIF3d and the 5′ m^7^G terminal cap structure, contributing to the minor differences observed between the measured binding affinities.

**Table 1:** Parameters describing the equilibrium binding of proteins to m^7^G capped CD101, ITGAE, and ACTB

	CD101			ITGAE			ACTB		
	Kd ± SD (nM)	Amp (r_max-_ r_min_)	${\;x}^{2}$	Kd ± SD (nM)	Amp (r_max-_ r_min_)	${\;x}^{2}$	Kd ± SD (nM)	Amp (r_max-_ r_min_)	${\;x}^{2}$
eIF3d	17 ± 2	0.041	0.997	26 ± 3	0.062	0.994	35 ± 4	0.040	0.996
eIF3d+GDP	20 ± 3	0.038	0.996	31 ± 2	0.054	0.996	43 ± 2	0.042	0.997
eIF3d+m^7^GDP	NSB			NSB			NSB		
eIF3d+eIF4G2	9 ± 2	0.061	0.985	12 ± 2	0.081	0.995	20 ± 3	0.051	0.985
eIF3d+eIF4G2+m^7^GDP		NSB			NSB			NSB	
eIF4E	45 ± 2	0.042	0.996	39 ± 3	0.036	0.994	20 ± 2	0.074	0.997
eIF4E+4EBP1	22 ± 2	0.046	0.995	18 ± 3	0.040	0.993	9 ± 2	0.082	0.997
eIF4E+eIF4G2	51 ± 2	0.041	0.996	40 ± 2	0.039	0.997	21 ± 3	0.062	0.998
eIF4G2	NSB			NSB			NSB		

*K*d is the equilibrium dissociation constant, r_max_–r_min_ is the amplitude which indicates change in anisotropy and ${\;x}^{2}$ represents the goodness of fit. NSB: no significant binding.

### eIF3d binding is cap-dependent

To further investigate the cap-binding specificity of eIF3d, the mononucleoside diphosphates 7-methylguanosine diphosphate (m^7^GDP) and GDP were used in our fluorescence anisotropy-based assays. m^7^GDP serves as a cap analog whereas GDP does not (42, 43). In the presence of m^7^GDP, binding of eIF3d to each of the m^7^G capped transcripts was significantly impaired (Fig. 3 and Table 1). The presence of GDP, however, had little or no effect on binding to the m^7^G capped transcripts (Fig. 3 and Table 1). These results demonstrate that eIF3d binding to the transcripts is cap-dependent.

### eIF4G2 enhances eIF3d binding to m^7^GpppA capped mRNA

**Figure 4:**
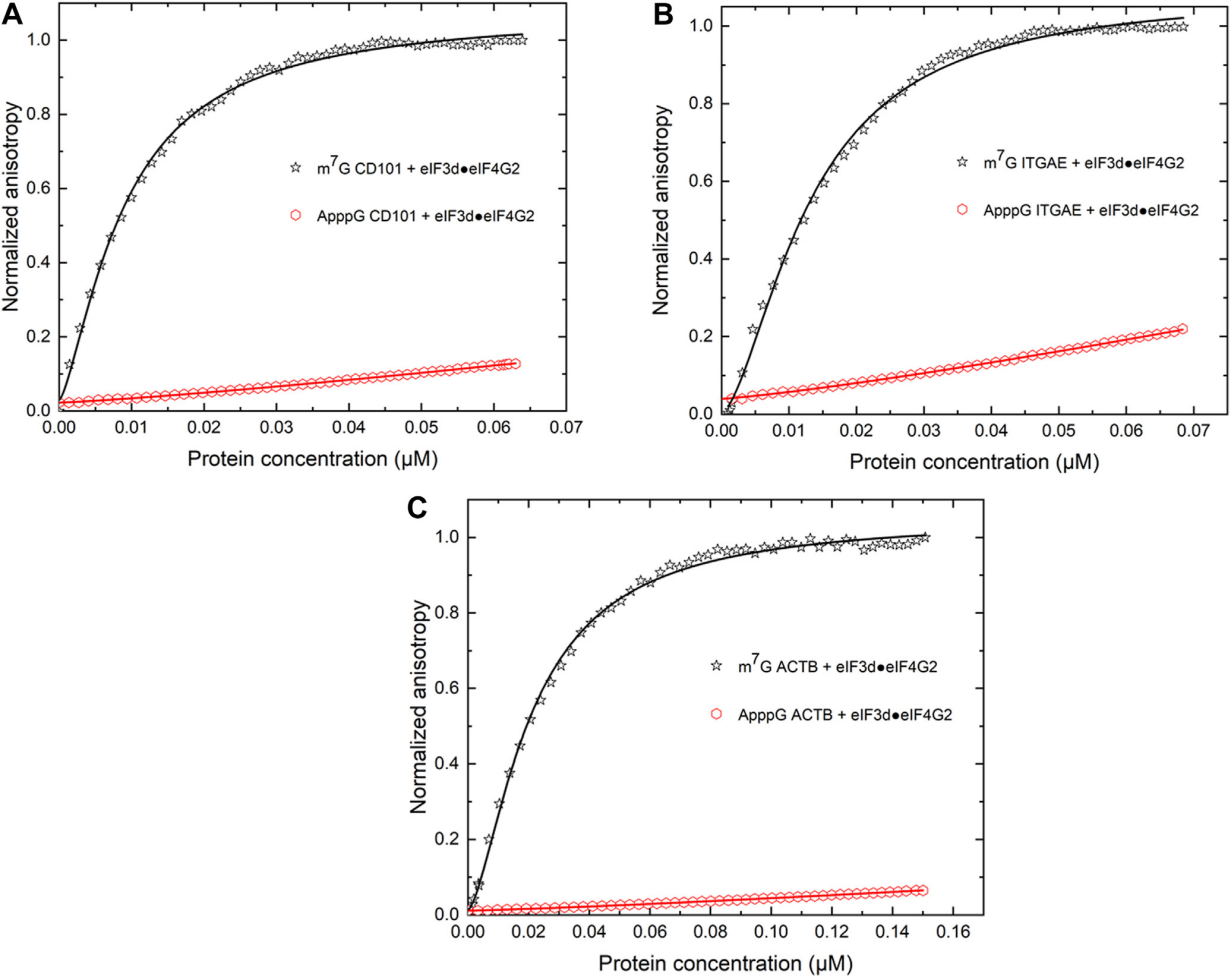
**Equilibrium-binding titrations of fluorescein-labeled m**^**7**^**G and ApppG capped 5′UTRs with the eIF3d and eIF4G2 complex.** Briefly, 10 nM of fluorescein-labeled m^7^G or ApppG capped 5′ UTRs of (*A*) CD101, (*B*) ITGAE, and (*C*) ACTB coding mRNAs were titrated with increasing concentration of protein/protein complex in the titration buffer at 25 °C. Saturating amounts of 5 μM eIF3d and 5 μM eIF4G2 protein/protein complex in the syringe were injected automatically into the cuvette containing the mRNA over a time course of 30 min. The anisotropy at each titration point was measured using excitation and emission wavelengths of 495 nm and 520 nm, respectively. Data points correspond to an average of three independent anisotropy measurements, and the curves represent the nonlinear fits that were used to obtain the averages and standard deviations for the corresponding K_d_ values presented in Table 1. ACTB, β-actin; eIF, eukaryotic initiation factor; CD101, cluster of differentiation 101; ITGAE, integrin subunit alpha E; m^7^G, 7-methylguanosine.

Recent findings indicate that eIF3d crosslinks to eIF4G2 (18). A direct interaction between eIF3d and eIF4G2 was confirmed by the use of live cell high-specificity bifunctional chemical crosslinking with bis(sulfosuccinimidyl) suberate (B3), stringent immunoprecipitation, and immunoblot analysis (18). However, a quantitative analysis of the influence of eIF4G2 on the cap binding activity of eIF3d was not previously elucidated. To gain insight into the effects of eIF4G2 on the cap recognition and binding activity of eIF3d, fluorescence anisotropy-based equilibrium assays were performed using protein-protein complexes of eIF3d and eIF4G2. Saturating amounts of eIF3d and eIF4G2 in the protein-protein complexes, corresponding to 5 μM of each protein component respectively were used. Analysis of equilibrium dissociation constants (K_d_'s) obtained from eIF3d/eIF4G2 complex binding to the m^7^G cap showed a 2-fold increase in binding affinity, compared to that of eIF3d alone for the 5′UTRs tested (Fig. 4 and Table 1). The probe (fluorescently labeled mRNA) concentrations used in these experiments were 10 nM. Thus equilibrium dissociation constants below 10 nM cannot be reliably determined. Therefore, the reason for these small changes in the binding affinities may not completely reflect changes in the affinity since binding is already very tight. Our fluorescence anisotropy-based equilibrium assays in this case are also limited by the concentrations of eIF3d and eIF4G2 in the protein-protein complexes. We only consider the eIF3d and eIF4G2 concentrations in the range that do not cause any abnormal anisotropy changes due to aggregation or precipitation.

To further ascertain whether the eIF3d/eIF4G2 complex specifically recognized the m^7^G cap, fluorescently labeled ApppG capped 5′UTRs were included in the assays. The eIF3d/eIF4G2 complex did not show any significant binding to the ApppG capped 5′UTRs (Fig. 4). In addition, a significant reduction in the binding affinity of the eIF3d/eIF4G2 complex to the m^7^G cap was also observed in the presence of m^7^GDP (Fig. 3 and Table 1). Altogether, these results indicate that the m^7^G cap structure is specifically recognized by the eIF3d/eIF4G2 complex and that binding is largely cap-dependent.

### CD101 and ITGAE encoding mRNAs preferentially bind to eIF3d and eIF4G2

Co-immunoprecipitation studies have recently demonstrated that a subset of mRNAs that depend on the cap-binding activity of eIF3d are able to also interact with eIF4E *via* their 5′ caps (44). Most importantly, the interaction with eIF3d and eIF4E does not happen simultaneously. During cellular conditions where eIF4E is sequestered, these mRNA subsets release eIF4E from their 5′ caps and bind to eIF3d instead. This shift to eIF3d binding is thought to follow a “let-go” mechanism (44).

**Figure 5:**
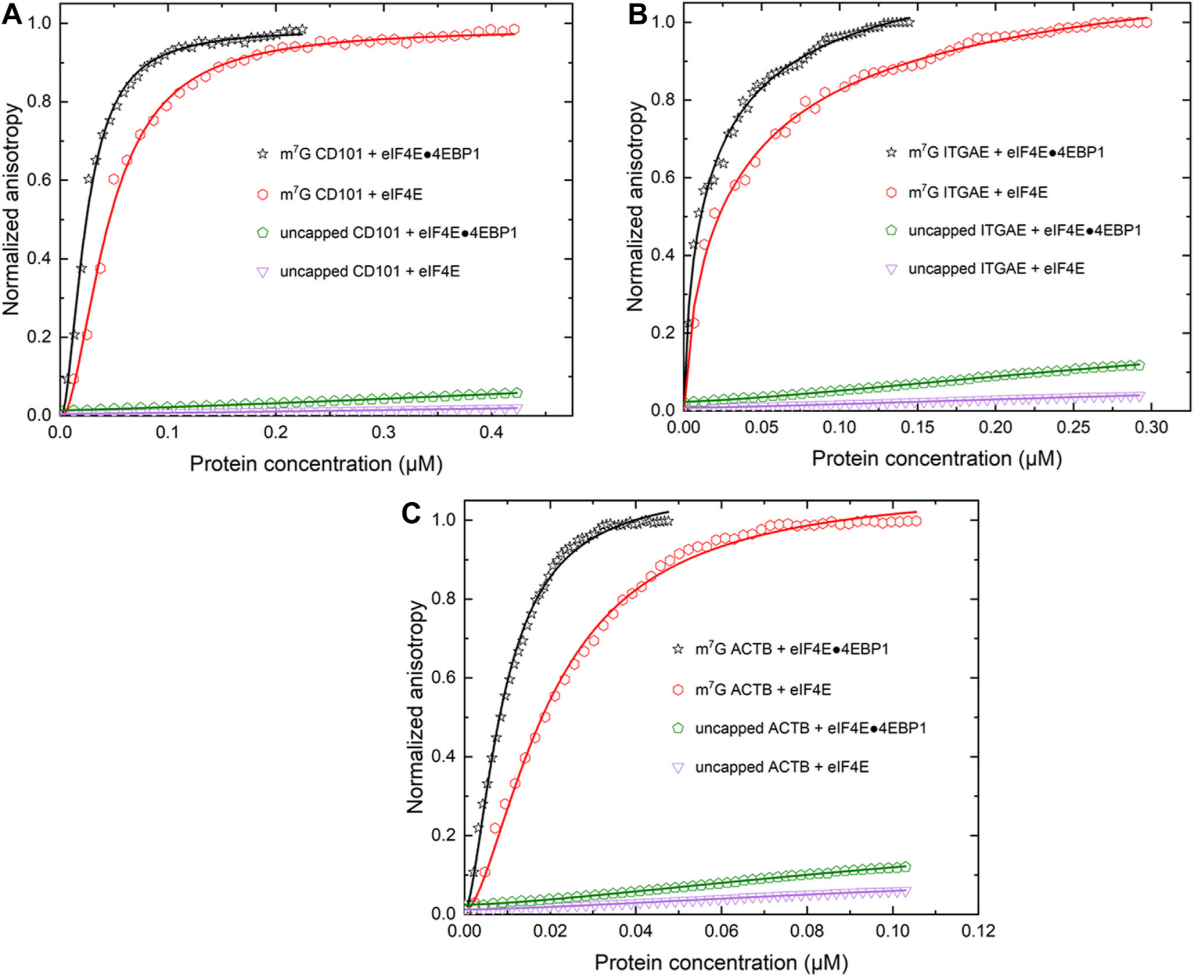
**Comparison of fluorescein labeled m**^**7**^**G capped or uncapped 5′UTRs binding with eIF4E or a complex of eIF4E and 4EBP-1**. Equilibrium-binding titrations of fluorescein-labeled m^7^G capped 5′UTRs of mRNAs coding for (*A*) CD101, (*B*) ITGAE, and (*C*) ACTB with either eIF4E and 4EBP-1 complex, or eIF4E alone are shown. The normalized anisotropy changes for the interaction between the fluorescein-labeled, but uncapped, 5′UTRs with the eIF4E and 4EBP-1 complex, or eIF4E alone were included as internal controls to allow comparisons of differing batches of RRL. Briefly, 10 nM of fluorescein-labeled capped or uncapped mRNAs were titrated with increasing concentration of protein or protein/protein complex in the titration buffer at 25 °C. The anisotropy at each titration point was measured using excitation and emission wavelengths of 495 nm and 520 nm, respectively. Data points correspond to an average of three independent anisotropy measurements. The curves represent the nonlinear fits that were used to obtain the averages and standard deviations for the corresponding K_d_ values presented in Table 1. 4EBP-1, 4E binding protein 1; ACTB, β-actin; CD101, cluster of differentiation 101; eIF4E, eukaryotic initiation factor 4E; ITGAE, integrin subunit alpha E; m^7^G, 7-methylguanosine; RRL, rabbit reticulocyte lysate.

In this regard, the affinities of the CD101 and ITGAE encoding mRNAs for eIF4E were determined (Fig. 5 and Table 1). The affinity of eIF4E for the 5′ UTR of ACTB encoding mRNA was also determined (Fig. 5 and Table 1). The measured equilibrium dissociation constants (K_d_)'s indicate that eIF4E bound the 5′ cap of ACTB encoding mRNA about 2-fold tighter (20 ± 2) nM than CD101 (45 ± 2) nM and ITGAE (39 ± 3) nM encoding mRNAs, respectively (Table 1). eIF4E did not show any significant binding to any of the uncapped transcripts, indicative of the vital role of the 5′ cap for eIF4E recruitment. Furthermore, 4EBP-1 bound eIF4E showed a 2-fold tighter binding to the m^7^G capped transcripts compared to eIF4E (Table 1). As anticipated, 4EBP-1-bound eIF4E did not show significant binding to the uncapped transcripts (Fig. 5).

Analysis of the equilibrium dissociation constants (K_d_)'s for the eIF3d/eIF4G2 complex binding to CD101 and ITGAE encoding mRNAs, compared to that of eIF4E alone binding to these mRNAs showed a 5-fold and 3-fold tighter binding, respectively, for the eIF3d/eIF4G2 complex than for eIF4E (Table 1). The measured equilibrium dissociation constant (K_d_) for 5′ capped CD101 encoding mRNA with the eIF3d/eIF4G2 complex was (9 ± 2) nM, and (45 ± 2) nM with eIF4E alone, respectively (Table 1). For 5′ capped ITGAE encoding mRNA on the other hand, the measured equilibrium dissociation constant (K_d_) was (12 ± 2) nM for the eIF3d/eIF4G2 complex and (39 ± 3) nM for eIF4E alone (Table 1). These results may be indicative of the dependence of CD101 and ITGAE encoding mRNAs on eIF3d and eIF4G2 as compared to eIF4E.

### eIF4G2 does not enhance the affinity of eIF4E for the m^7^G cap

To establish that eIF4G2 is specific for eIF3d, the effect of eIF4G2 on the affinity of eIF4E for the 5′ cap was investigated. To achieve this, the fluorescently labeled but 5′ capped 5′UTRs were titrated with eIF4E/eIF4G2 protein/protein mixtures. Saturating amounts of eIF4E and eIF4G2 in the mixtures, corresponding to 5 μM (μM) of each protein component, were used. As discussed earlier, the probe concentration used in these experiments was also 10 nM.

**Figure 6:**
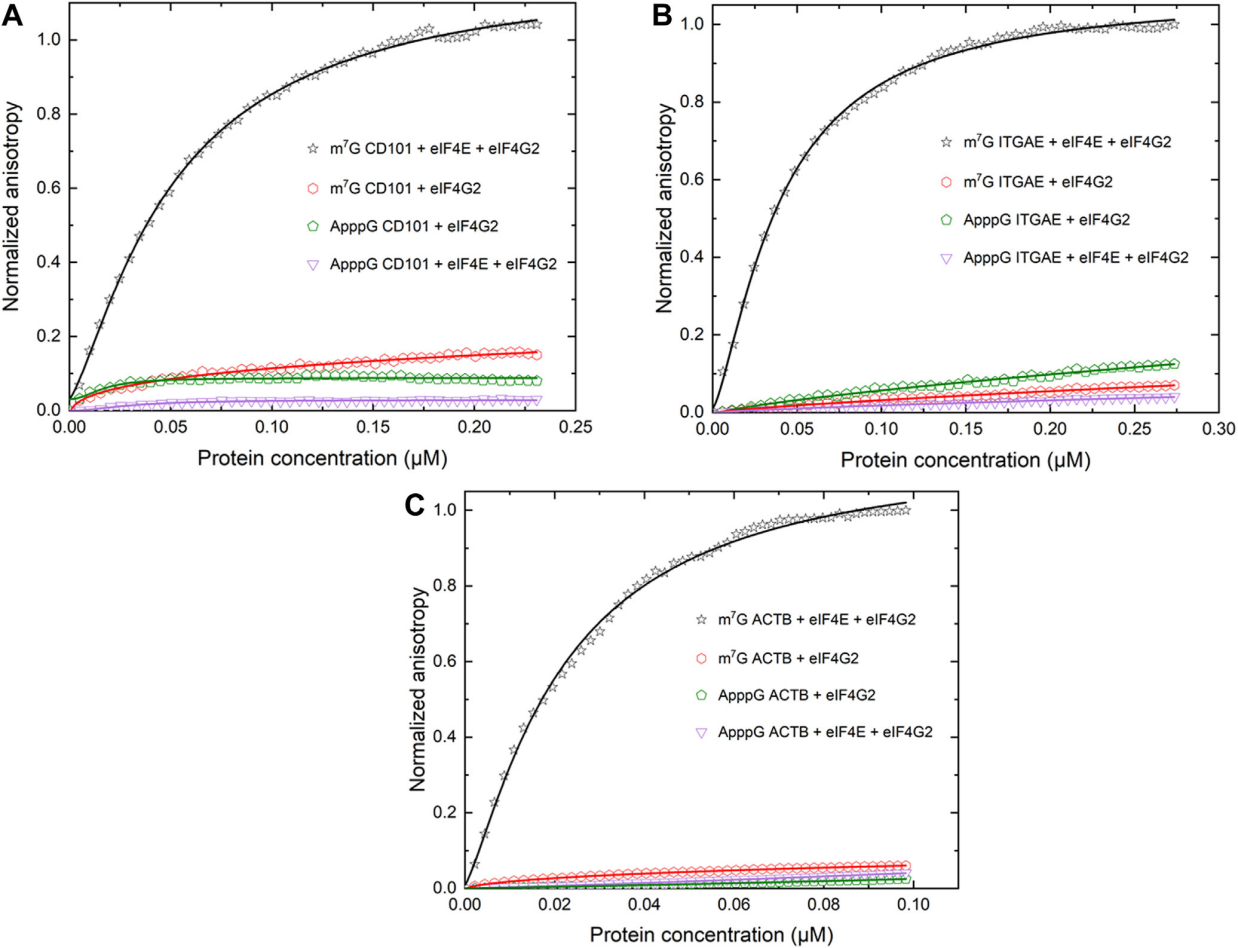
**Effect of eIF4G2 on the cap-binding activity of eIF4E.** Equilibrium-binding titrations of fluorescein-labeled m^7^G and ApppG capped 5′UTRs of mRNAs coding for (*A*), CD101, (*B*), ITGAE, and (*C*) ACTB with either eIF4E and eIF4G2 protein/protein mixtures, or eIF4G2 alone. Briefly, 10 nM of fluorescein-labeled m^7^G or ApppG capped mRNAs were titrated with increasing concentration of protein or protein/protein mixtures in the titration buffer at 25 °C. The anisotropy at each titration point was measured using excitation and emission wavelengths of 495 nm and 520 nm, respectively. Data points correspond to an average of three independent anisotropy measurements, and the curves represent the nonlinear fits that were used to obtain the averages and standard deviations for the corresponding K_d_ values presented in Table 1. ACTB, β-actin; CD101, cluster of differentiation 101; eIF4E, eukaryotic initiation factor 4E; ITGAE, integrin subunit alpha E; m^7^G, 7-methylguanosine.

Analysis of the equilibrium dissociation constants (K_d_)'s revealed that eIF4G2 did not further improve the binding of eIF4E for the 5′ cap (Fig. 6 and Table 1). The affinity of eIF4E for the 5′ cap remained largely unaffected in the presence of eIF4G2 (Fig. 6 and Table 1). eIF4E/eIF4G2 protein/protein mixtures did not bind to the ApppG capped transcripts, indicative of the pivotal role of a fully matured methylated cap structure for recruitment and recognition (Fig. 6).

To further test if the observed anisotropy changes were solely as a result of eIF4E binding, the affinities of eIF4G2 for all the 5′UTRs in both the m^7^G and ApppG capped forms were also determined. eIF4G2 alone did not show appreciable binding to any of the 5′UTRs tested, irrespective of whether they were m^7^G or ApppG capped (Fig. 6 and Table 1). eIF3d/eIF4G2 binding to 5′ capped CD101 and ITGAE encoding mRNAs also show a 5-fold and a 3-fold tighter binding respectively, compared to that of eIF4E/eIF4G2 binding to the same transcripts (Table 1). Altogether, these results emphasize two major points. The first being that eIF4G2 is much more specific for eIF3d, and the second being that eIF4G2 is more dependent on the cap-binding activity of eIF3d rather than eIF4E.

### CD101, ITGAE, and ACTB encoding mRNAs are translated in a cap-dependent manner

In order to gain a better understanding of the translational activities of CD101, ITGAE, and ACTB encoding mRNAs, *in vitro* translation assays were performed using nuclease treated rabbit reticulocyte lysates (RRLs).

**Figure 7:**
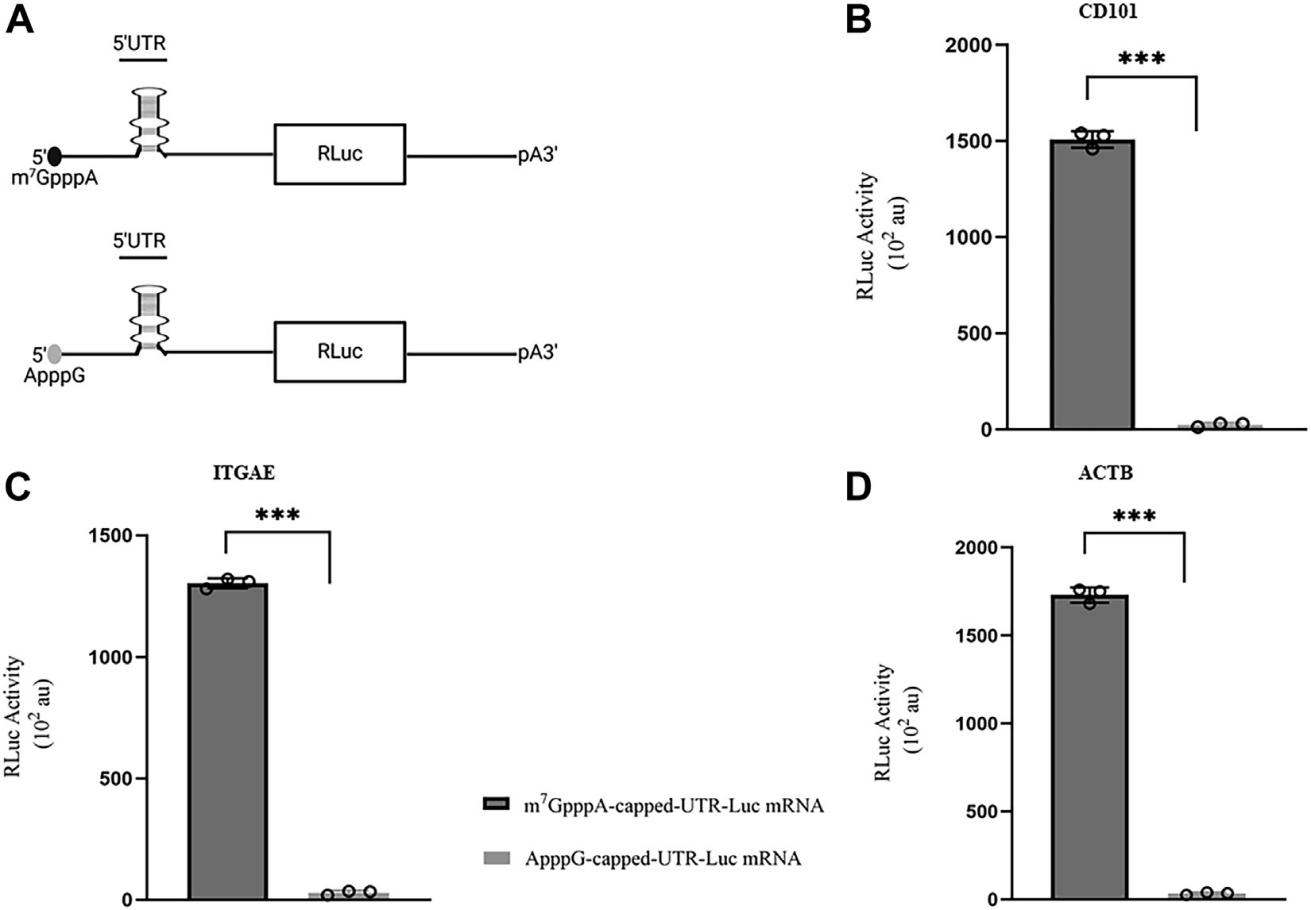
**Translational efficiencies of m**^**7**^**GpppA and ApppG capped-UTR-Luc mRNAs.***A*, schematic showing the m^7^GpppA and ApppG reporter constructs. Translational outputs of m^7^GpppA and ApppG capped 5′ UTR-luc-mRNAs (*B*) CD101, (*C*) ITGAE, and (*D*), ACTB. Bar heights and error bars correspond to the average and standard deviations, respectively, of three independent luciferase activity measurements. Data were analyzed by two-tailed unpaired student's *t* test; ∗∗∗, *p* < 0.001. ACTB, β-actin; CD101, cluster of differentiation 101; ITGAE, integrin subunit alpha E; m^7^G, 7-methylguanosine.

First, the effect of either an m^7^GpppA or an ApppG 5′ end modification on the absolute translation levels of CD101, ITGAE, and ACTB encoding mRNAs in the lysates was investigated (Fig. 7*A*).

For CD101 encoding mRNA, translational yield was reduced to 1.4% with the incorporation of a non-functional ApppG cap (Fig. 7*B*). Similarly, translational yield for the ITGAE encoding mRNA was significantly reduced to just 2.1% with the incorporation of the non-functional ApppG cap (Fig. 7*C*). The yield of translation for the ACTB encoding mRNA also dropped to 1.9% when the 5′ end modification of the mRNA was an ApppG cap (Fig. 7*D*). Altogether, these results indicate that the CD101, ITGAE, and ACTB encoding mRNAs are preferentially translated in a cap-dependent manner, with a strong reliance on the m^7^GpppA cap.

### eIF3d and eIF4G2 promote the translation of CD101 and ITGAE encoding mRNAs

**Figure 8:**
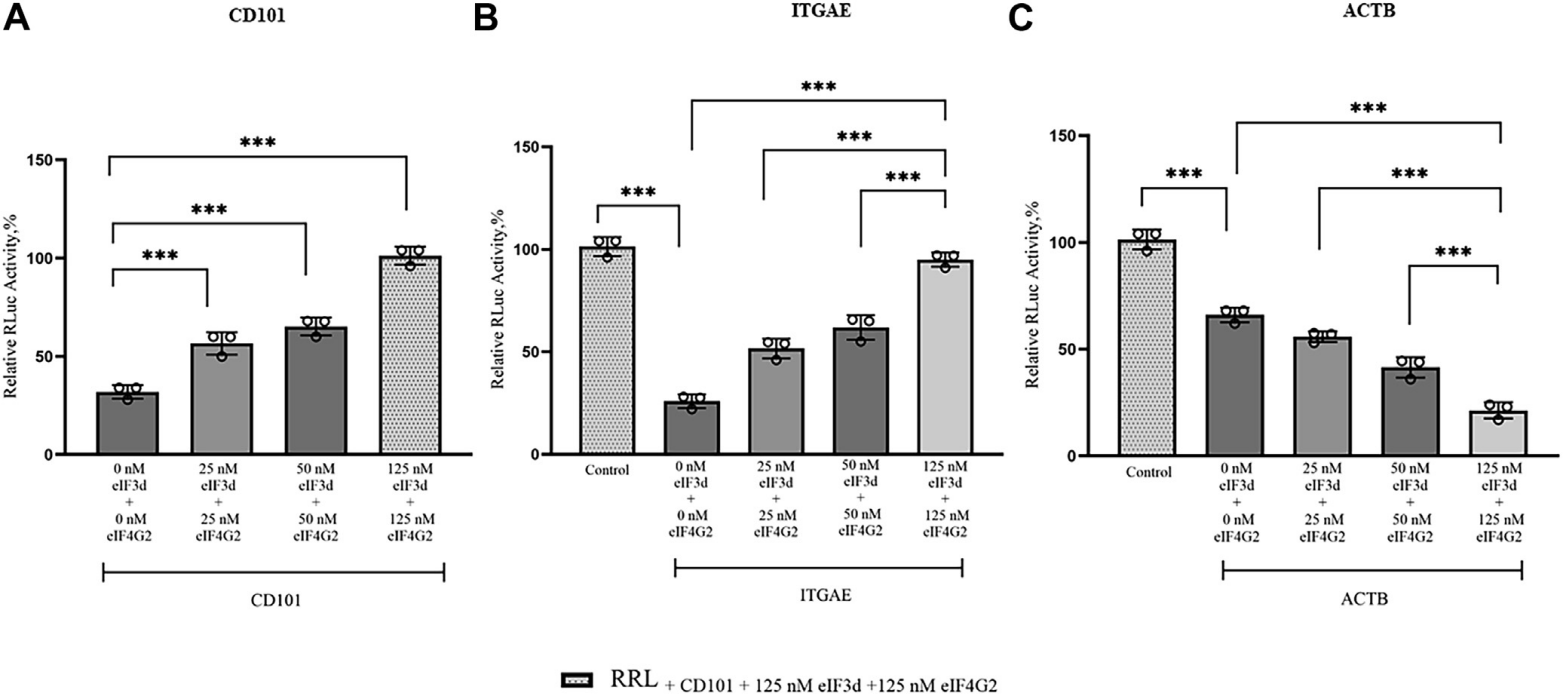
**Effect of increasing concentrations of eIF3d and eIF4G2 on the translational yields of m**^**7**^**GpppA capped transcripts.** Panels (*A*–*C*) represent the CD101, ITGAE, and ACTB 5′ UTR encoding mRNA luciferase constructs, respectively. Luciferase activity was normalized to a control eIF3d dependent mRNA, CD101. Luciferase activity measured in RRL for CD101 with the addition of the 125 nM eIF3d/eIF4G2 protein mixture was set at 100% and used as an internal positive control. Bar heights and error bars correspond to the average and standard deviations, respectively, of three independent luciferase activity measurements. Data were analyzed by two-tailed unpaired Student's *t* test; ∗∗∗, *p* < 0.001. ACTB, β-actin; CD101, cluster of differentiation 101; eIF4E, eukaryotic initiation factor 4E; ITGAE, integrin subunit alpha E; m^7^G, 7-methylguanosine; RRL, rabbit reticulocyte lysate.

It has been reported that under conditions of stress where the eIF3d-dependent mechanism of translation occurs, there is the overexpression of eIF3d and eIF4G2 (20, 41, 45). Thus the effect of an increase in the concentration of eIF3d and eIF4G2 on the translation of m^7^GpppA CD101, ITGAE, and ACTB encoding mRNAs was tested. eIF3d and eIF4G2 favored the translation of CD101 and ITGAE encoding mRNAs (Fig. 8, *A* and *B*). Surprisingly, for ACTB encoding mRNA, despite binding of eIF3d and eIF4G2 to the capped mRNA, an inhibitory effect on translation was observed when these proteins were added (Figs. 4 and 8*C*).

### ACTB encoding mRNA is more dependent on eIF4E for translation

**Figure 9:**
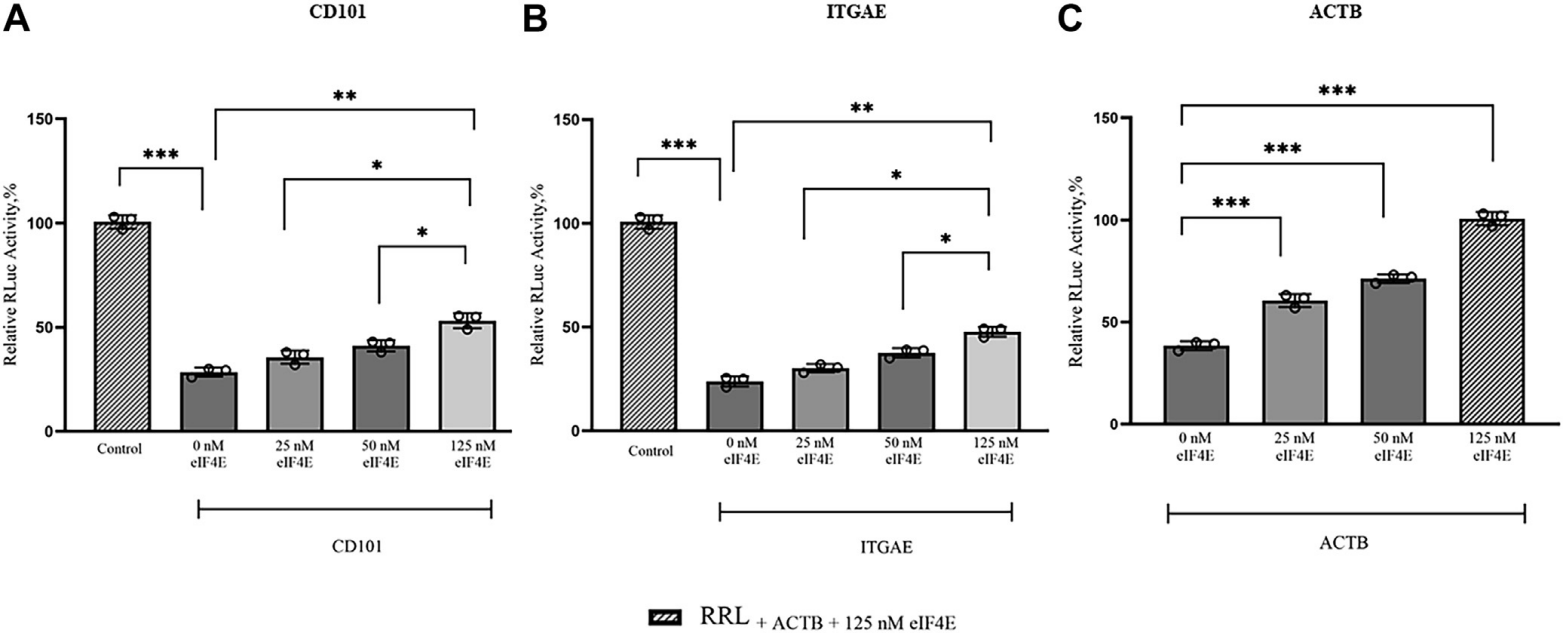
**Effect of increasing concentrations of eIF4E on the translational outputs of m**^**7**^**GpppA cappedtranscripts .** Panels (*A*–*C*), represent the 5′ UTR luc-mRNAs encoding CD101, ITGAE, and ACTB, respectively. Luciferase activity was normalized to a control eIF4E dependent mRNA, ACTB. Luciferase activity measured in RRL for ACTB with the addition of 125 nM eIF4E was set at 100% and used as an internal positive control. Bar heights and error bars correspond to the average and standard deviations, respectively, of three independent luciferase activity measurements. Data were analyzed by two-tailed unpaired student's *t* test; ∗*p* < 0.033, ∗∗*p* = 0.002; ∗∗∗*p* < 0.001. 4E; ITGAE, integrin subunit alpha E; ACTB, β-actin; CD101, cluster of differentiation 101; eIF4E, eukaryotic initiation factor m^7^G, 7-methylguanosine; RRL, rabbit reticulocyte lysate.

Moreover, the effect of an increase in the concentration of added eIF4E to the translation of CD101, ITGAE, and ACTB encoding mRNAs was also investigated. eIF4E favored the translation of the ACTB encoding mRNA much more, compared to CD101 and ITGAE encoding mRNAs (Fig. 9). For example, at 125 nM added eIF4E the translational yield of the CD101 encoding mRNA was 52% relative to the control ACTB encoding mRNA (Fig. 9*A*). At the same concentration of added eIF4E (125 nM), the translational yield of the ITGAE encoding mRNA was 47% relative to the control ACTB encoding mRNA (Fig. 9*B*), whereas at only 25 nM added eIF4E, translational yield of the ACTB encoding mRNA was already at 60% (Fig. 9*C*). Contrary to the inhibitory effect observed with ACTB encoding mRNA in the case of increasing concentration of added eIF3d and eIF4G2 (Figs. 8*C* and S2), we did not observe inhibitory effects with eIF4E addition on any of the mRNAs tested. The inhibitory effect of eIF3d and eIF4G2 observed with the ACTB encoding mRNA was, however, overcome with the addition of purified exogenous eIF4E (Fig. S2).

### Effects of 4EBP-1 on eIF3d/eIF4G2 and eIF4E mediated cap-dependent translation

Having determined that the translation of CD101 and ITGAE encoding mRNAs proceeds preferentially in an eIF3d and eIF4G2 dependent manner, and that of ACTB in an eIF4E dependent manner, the effect of 4EBP-1 on translational activity was investigated. 4EBP-1 inhibits eIF4E-eIF4GI interactions. 4EBP-1 did not suppress the eIF3d and eIF4G2 dependent translation of CD101 and ITGAE encoding mRNAs (Fig. S3). However, an increase in the concentration of 4EBP-1 led to a suppression of the translational activity of the ACTB encoding mRNA (Fig. S3).

## Discussion

It has long been known that most cellular mRNAs are translated *via* the eIF4E cap-dependent mechanism (1, 5, 7). Although alternate translational mechanisms exist, such as internal ribosome entry site directed translation which is independent of eIF4E and mTORC1 activity, only 3% - 5% of cellular mRNAs are thought to employ this mechanism (17). Recent studies reveal that in spite of quantitative silencing of eIF4E, and its sequestration by 4EBP-1 as a result of mTORC1 inhibition, there is still translation of about 20% or more capped mRNAs in the cell (17). This suggests that in the cell, these capped mRNAs are able to bypass the canonical eIF4E requirement, and translate in an eIF4E independent manner (17, 41). Although the varying mechanisms of translation in which eIF4E is dispensable have not been fully understood yet, of interest is the eIF3d and eIF4G2 mediated form of translational switch and reprogramming. While earlier studies have attempted to decipher this important mechanism of translational control, there has not yet been any quantitative study aimed at addressing how eIF3d or eIF3d and eIF4G2 complexes are recruited to drive this alternate form of cap-dependent translation initiation.

Here, we report the first quantitative and mechanistic studies of a novel translation initiation mechanism which utilizes eIF3d, a subunit of the large protein complex eIF3. In this study, by utilizing the fluorescence anisotropy-based equilibrium assay, we show the direct binding of eIF3d to a subset of human mRNAs. Our biophysical studies emphasize the important role of the m7G cap structure in the recruitment of eIF3d (Fig. 3). The significant reduction in the cap binding ability of eIF3d observed in the presence of competitor ligands m^7^GDP, but not GDP is indicative of the need for a fully methylated 5′ cap structure for eIF3d recognition and subsequent interaction with the mRNA (Fig. 3 and Table 1).

eIF3d complexed with eIFG2 exhibited a much stronger cap-recognition ability. This was most prominent among the eIF3d dependent mRNAs, CD101, and ITGAE, which also function as important regulatory T-cell markers. In regulatory T-cells for example, the eIF3d and eIF4G2 mediated translational switch has been utilized in maintenance, survival, and cell invasion (17, 20, 41). In certain types of cancers, the eIF3d and eIF4G2 driven mechanism is also used to bypass the global sequestration of eIF4E, thereby allowing EMT, which is a crucial indicator for cancer progression to occur (17). It is also important to note that the eIF3d and eIF4G2 complex specifically recognized the m^7^G cap structure, as replacement of the m^7^G cap with an ApppG cap structure, resulted in no binding to the mRNA transcripts (Fig. 4). These observations further bolster the critical role which the fully methylated 5′ cap structure plays in the recruitment of the eIF3d and eIF4G2 complex.

In addition, the significant reduction in the binding activity of the eIF3d and eIF4G2 complex to the cap in the presence of m^7^GDP may indicate that the complex is favorably oriented in such a way that the cap binding domain of eIF3d specifically interacts with the m^7^G cap structure. A blockage of this cap binding site by the analogue, subsequently, prevents the complex from interacting with the mRNA cap.

Our studies indicate that CD101 and ITGAE encoding mRNAs preferentially bind to the eIF3d and eIF4G2 complex much better than eIF4E. Recent coimmunoprecipitation studies by *Roiuk et al.* (44), show that during mTORC1 inhibition, mRNAs that depend on eIF3d decrease their binding to eIF4E, but increase their binding to eIF3d and eIF4G2. These mRNAs are able to “let go” of eIF4E and bind to eIF3d and eIF4G2 enabling them to be translated (44). Thus the 5-fold and 3-fold differences observed in the binding of CD101 and ITGAE encoding mRNAs for the eIF3d and eIF4G2 complex as compared to eIF4E, may be essential for the quick release of eIF4E from their caps, and the subsequent binding to the eIF3d and eIF4G2 complex to promote translation. This may also depend on the cellular concentrations and availability of these components, as well as additional factors that may be required for stabilization or release of eIF4E.

Moreover, our quantitative studies show the specificity of eIF4G2 for the cap-binding protein eIF3d but not eIF4E (Figs. 4 and 6). Whereas eIF4G2 enhanced the affinity of eIF3d for the 5′ cap, eIF4G2 did not improve the binding of eIF4E for the 5′ cap structure (Fig. 6 and Table 1). These observations highlight the cooperativity that exists between eIF3d and its binding partner eIF4G2 in mediating this alternative form of cap-dependent translation, correlating with earlier research from the Cate group (21, 22).

The specificity of eIF3d and eIF4G2 for the translation of CD101 and ITGAE encoding mRNAs, but not ACTB encoding mRNA, in the *in vitro* translation assays also shed more light on the initiation factor preferences of the mRNA transcripts (Figs. 8 and 9). eIF3d has been known to be highly selective, and required in driving the specialized translation of only specific mRNA subsets (21, 22, 41). Notably, how eIF3d and eIF4G2 identify and discriminate between different mRNA groups remains poorly understood (18, 20). The inhibitory effect observed on the translation of ACTB encoding mRNA in the presence of eIF3d and eIF4G2, may be due to the obstruction of eIF4E's free access to the 5′ cap by eIF3d and eIF4G2. Since ACTB is strongly dependent on eIF4E, this obstruction may account for the significant drop in the levels of translation (Fig. 8). It is noteworthy, that this drop in translation is overcome with the addition of free exogenous eIF4E, indicating that eIF4E may be able to compete for access to the 5′ cap in such cases (Fig. S2). eIF3d has been known to either promote or repress the translation of different mRNA subgroups. In *Drosophila*, for example, eIF3d and its binding partner Hrp48 have been shown to repress the translation of the msI-2 mRNA (46). Furthermore, although 4EBP-1 inhibited the translation of ACTB encoding mRNA, eIF3d and eIFG2 still allowed for the translation of CD101 and ITGAE encoding mRNAs specifically, thereby overcoming the inhibitory effects of 4EBP-1(Fig. S3). This observation indicates that specific mRNA subsets are able to translate by utilizing eIF3d and eIFG2 under conditions where eIF4E is inhibited. These findings are also consistent with recent work done by *Roiuk et al.*, (44).

In closing, the question arises as to the extent to which the eIF3d subunit can function independent of the other eIF3 proteins. First, recent work done by De La Parra *et al.* (41), using STRING analysis shows that, unlike the interaction between eIF3d and eIFGI which is weak and indirect *via* other eIF3 proteins, the interaction between eIF3d and eIF4G2 is direct. Secondly, *Hayek et al.* (47), performed GST pull-down experiments using total RNA from HEK293FT cells and recombinant HisGST eIF3c, d, e, and g proteins to test their ability to interact directly and independently of the eIF3 complex with histone mRNAs. The results of their studies showed that the eIF3d subunit was able to interact with histone mRNAs independently of the eIF3 complex, confirming earlier cross-linking data (47). In addition, a recent review by Ma *et al.* (25), reports that the function of eIF3d in regulating the noncanonical translation of specific mRNA subsets can also proceed independent of the eIF3 complex. Furthermore, this review also states that eIF3 subunits may also have multiple specific noncanonical functions to activate or repress translation of different subgroups of mRNAs independent of the eIF3 complex (25). *Masutani et al.* (48, 49), as well as the Cate group found that the d-subunit of eIF3 was structurally dispensable for the assembly of the remaining eIF3 subunits. Finally, the Valášek group also published findings that similar to eIF3j, eIF3d is the only subunit whose knockdown affects neither the protein levels of the other eIF3 subunits nor the integrity of the eIF3 complex (50).

Research on how eIF3d is able to drive noncanonical translation in a manner independent of eIF4E is still emerging and holds great promise for the future. Although our studies specifically focus on the eIF3d subunit and eIF4G2, future work directed at also studying how eIF3d in the context of the whole eIF3 complex, as well as interactions it makes as part of the 43S preinitiation complex may be of interest.

In sum, this study provides the first quantitative insight into the recruitment of eIF3d as well as eIF3d and eIF4G2 complexes to 5′ capped mRNA—an alternative strategy to drive the cap-dependent translation of a subset of human mRNAs without the need for eIF4E. This study lays the foundation for a quantitative understanding of eIF3d driven translation to guide in the design and development of novel therapeutic targets in cancer research.

## Experimental procedures

### Preparation of RNAs for fluorescence anisotropy-based equilibrium binding studies

DNA templates corresponding to the 5′UTRs of mRNAs encoding CD101 (66 nucleotides, GenBank accession number XM_054339676.1, ITGAE (99 nucleotides, GenBank accession number L25851.2) and ACTB (84 nucleotides, GenBank accession number AK301372.1), respectively (Table S1) were purchased from Integrated DNA Technologies, and the corresponding RNAs were synthesized *via in vitro* transcription using the HiScribe T7 Quick High Yield RNA Synthesis Kit (New England Biolabs Inc) following the manufacturer's protocol (38, 51). Purified RNA transcripts were then labeled at the 3′ termini in an oxidation reaction using a final concentration of 30 μM RNA, 105 μM of NaOAc pH-5.2, and 15 mM of sodium periodate. The reaction tube was covered with aluminum foil and incubated for 30 min in the dark (38, 51). After incubation, 1M sodium sulphite was added and incubated for an additional 10 min in the dark. The oxidation product was purified using ethanol precipitation. The purified oxidized RNA was then used for a labeling reaction by adding a final concentration of 1 mM flouroscein-5-thiosemicarbazide and 50 mM Na-phosphate buffer pH 6.5 (38). The reaction was covered with aluminum foil and incubated in the dark for 2 h. After the incubation period, 1 M NaCNBH_3_ was added, and the reaction was further incubated overnight at 4 °C. After overnight incubation, the labeled product was purified using a two-step purification that involves ethanol precipitation and the use of the RNA Clean and Concentrator Kit from Zymo Research, following the manufacturer's protocol. The now purified and labeled RNAs were then capped using the vaccinia capping system, catalog number M2080S from New England Biolabs. The RNA concentrations were determined using nano-drop UV-visible spectrometer and integrity was verified by 1.5% agarose gel electrophoresis (38).

### Purification of eIF4G2, eIF3d, eIF4E, and 4EBP-1

The plasmid encoding full-length human eIF4G2 with an N-terminal 6x-histidine tag was purchased from GenScript. The eIF3d clone was a generous gift from Prof. Christopher S. Fraser (UC Davis). Human 4EBP-1 protein sequence was obtained from NCBI protein data bank (GenBank: BC058073.1). 4EBP-1 corresponding nucleotide sequence was subcloned into the NdeI and XhoI sites of a modified pET28a-TEV expression vector. The plasmid encoding full-length 4E-BP1 with an N-terminal 6× histidine tag was purchased from GenScript. Plasmid encoding 4EBP-1 was transformed into *Escherichia coli* BL21 (DE3) competent cells using the New England Biolabs Transformation protocol.

All the proteins were recombinantly expressed in *E. coli BL*21- CodonPlus (DE3)-RIL cells (Agilent) and were purified using a combination of nickel-nitrilotriacetic acid (Ni-NTA) affinity and heparin affinity columns, as previously described (38). Briefly, the proteins were first purified from bacterial cell lysates using His-Trap HP (Ni-NTA) columns (GE HealthCare Life Sciences), as per the manufacturer's instructions (38, 51). The purified 6x-histidine tagged proteins were dialyzed overnight against storage buffer (20 mM Hepes–KOH pH 7.6, 200 mM KCl, 10 mM β-mercaptoethanol, and 10% glycerol) in the presence of tobacco etch virus (TEV) protease to cleave off the tags. The untagged proteins were further purified and concentrated using 1 ml HiTrap Heparin HP columns (GE HealthCare Life Sciences) (38). The eluted proteins were analyzed on 10% SDS-PAGE gels, and pure fractions (>95% purity) were pooled and dialyzed overnight against storage buffer. The concentrations of the purified, concentrated proteins were quantified using Coomassie Protein Assay Reagent (Thermo Fisher Scientific) and were aliquoted and stored at −80 °C (38, 51).

The human eIF4E clone was also a kind gift from Prof. Christopher S. Fraser (UC Davis). This eIF4E construct had an N-terminal polyhistidine tag, with a TEV protease cleavage site between the histidine tag and eIF4E. For expression of recombinant eIF4E protein, *E. coli* Rosetta (DE3) was transformed with this clone and bacterial cells from 1.5 L of LB medium were used. The bacterial cells were grown at 37 °C till the *A*_600_ reached 0.6 to 0.8. Protein expression was induced overnight at 20 °C, by adding IPTG (final concentration of 0.5 mM). The cells were pelleted and sonicated in the lysis buffer (25 mM Hepes pH 7.5, 300 mM KCl, 10% glycerol, 1 mM DTT, 20 mM imidazole, and protease inhibitor tablet). The supernatant was filtered through a 400-micron syringe filter, and loaded on a pre-equilibrated 5 ml Ni-NTA column (pre-equilibrated with the lysis buffer). The column was washed with 20 ml buffer E (25 mM Hepes pH 7.5, 300 mM KCl, 10% glycerol, 1 mM DTT, and 50 mM imidazole), and the protein was eluted with buffer E containing 500 mM imidazole. Fractions containing eIF4E were pooled and dialyzed overnight against buffer E (without any imidazole) containing TEV protease, at 4 °C. The dialyzed samples were further purified on a 5 ml Q-Sepharose column to separate the untagged protein from the cleaved tag and the TEV protease. The KCl concentration of dialyzed samples was adjusted to 100 mM, and the sample was loaded onto a 5 ml preequilibrated Q-Sepharose column (preequilibrated with buffer A-25 mM Hepes pH 7.5, 100 mM KCl, 10% glycerol, and 1 mM DTT). The proteins were eluted using a step gradient of 150 to 500 mM KCl in buffer A. Fractions containing eIF4E, were pooled, concentrated, and stored at −80 °C. The protein concentrations were estimated using the Bradford's assay.

### Fluorescence anisotropy-based equilibrium binding assays

Fluorescein-labeled RNAs were diluted to 10 nM using buffer with constituents as follows; 20 mM Hepes–KOH, pH 7.5, 100 mM KCl, and 1 mM MgCl_2_. Fluorescence anisotropy measurements were performed using the equilibrium titration module of an SF-300X stopped-flow fluorimeter (KinTek Corporation). Fluorescein-labeled RNAs were excited at 495 nm, and emission was detected using a 515 nm high-pass filter (Semrock) (38). Equilibrium binding titrations began with a 200 μl sample of 10 nM of fluorescein-labeled RNA in the titration buffer (20 mM Hepes–KOH, pH 7.5, 100 mM KCl, and 1 mM MgCl_2_) and 20 to 50 data points were collected for each anisotropy measurement by automated continuous injection of 20 μl of protein over a period of 30 min at a temperature of 25 °C. Note that the first reading is taken in the absence of protein (38). Using the Origin 2023 software package (https://www.originlab.com/2023), the data were fitted to a nonlinear, equilibrium binding equation of the form:

$$r_{\text{obs}}=r_{\min}+\left(r_{\max}-r_{\min}\right)\left[\frac{\left[\text{Protein}\right]}{K_{d}+\left[\text{Protein}\right]}\right]$$

where r_obs_ is the observed anisotropy value, r_min_ is the minimum anisotropy value in the absence of protein, and r_max_ is the final saturated anisotropy value. The concentration of protein is represented as [Protein] and K_d_ is the equilibrium dissociation constant. The chi-squared values (χ^2^) that represented the statistical goodness of fit were always close to 1. Fitting data to a two-site model did not improve the fit as judged by (χ^2^) values (38). The equilibrium binding titration of each mRNA with the various 5′ end modifications (either m^7^G capped, ApppG capped or uncapped) was performed three times and fit independently for K_d_. The fitted K_d_'s were then averaged, and the standard deviations were calculated (38).

### Preparation of UTR-Luc reporter mRNAs for luciferase-based gene expression assays

In order to generate linearized plasmid DNA templates for *in vitro* transcription, plasmid DNAs were linearized using AvrII restriction enzyme. The resulting linearized DNA was purified using the GeneJET gel extraction and DNA cleanup Micro Kit from GeneJET as per the manufacturer's instructions (38, 51). DNA templates were *in vitro* transcribed using T7 RiboMAX Large Scale RNA Production Kit (Promega) following the manufacturer's protocol (38, 51). ApppG (NEB) or Ribo m^7^GpppA Cap Analog (Promega) was added to the transcription mix in an ApppG or m^7^GpppA: GTP ratio of 10:1 to get mRNA transcripts with either nonfunctional, or functional caps, respectively. Capped mRNAs were polyA tailed (pA) using the Poly(A) Tailing Kit (NEB, Cat# M0276) following the manufacturer's protocol. The resulting capped and polyadenylated mRNAs were then purified using RNA Clean and Concentrator Kit (Zymo) following the manufacturer's protocol (38, 51). RNA concentrations were determined using nano-drop UV/Vis spectrometer and integrity was verified by 1.0% agarose gel electrophoresis.

### Luciferase-based gene expression reporter assays

Gene expression was achieved by translating the mRNAs *in vitro*, using the nuclease treated RRL *in vitro* translation system from Promega. Each 25 μl reaction contained 70% v/v of RRL (Promega) supplemented with 0.5 mM MgCl_2_, 0.02 mM amino acid mixture, 10 U/μl RiboLock RNase Inhibitor (Thermo Fisher Scientific), and varying concentrations of purified protein. The RRL was made more cap-dependent by the addition of 75 mM KCl (38, 51). Briefly, 1 μg (60 nM) of UTR-Luc mRNA was added to the RRL *in vitro* translation mixture following the addition of the specified concentration of protein. The resulting *in vitro* translation reaction was then incubated at 30 °C for 1 h after which the reaction was stopped by placing the tubes on ice (38, 51). Luciferase activities were then assayed using SpectraMax iD5 Multi-Mode Microplate reader (Molecular Devices). Prior to taking luminescence measurements in the illuminometer, the samples were prepared by adding 5 μl of the translation reaction to 40 μl of Renilla-Glo Luciferase assay reagent (Promega). Readings in the illuminometer were taken over a spectral wavelength range of 350 to 650 nm and an integration time of 10s at room temperature. After subtracting the background, measured using an *in vitro* translation reaction to which no UTR-Luc mRNA had been added, the luminescence data were analyzed and plotted using the Graph Pad Prism 9 software package  (https://www.graphpad.com). At least three different batches of RRLs were used (38, 51). The translation data for each UTR-Luc mRNA were reported as an average of three independent experiments (38, 51). Each independent experiment was done in triplicates and the mean ± SD was calculated using the GraphPad Prism 9 software. Statistical significance between the mean values was analyzed using two-tailed unpaired student's *t* test (GraphPad Prism 9 software). The statistical significance was set at *p* < 0.05 with corresponding *p*-values calculated. The calculated *p*-values are indicated on the brackets above the bar graphs, and in the figure captions, respectively.

## Data availability

All data are present in the manuscript or supplementary data.

## Supporting information

This article contains supporting information.

## Conflict of interest

The authors declare that they have no conflicts of interest with the contents of this article.

## Figures and Tables

**Supplementary Figure 1:**
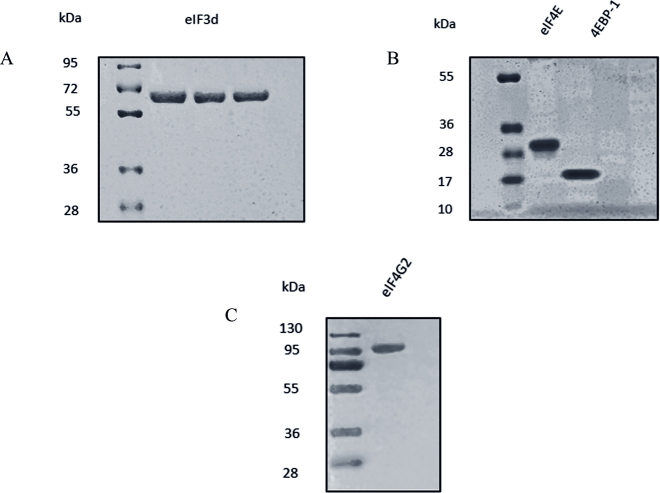


**Revised supplementary Figure 2:**
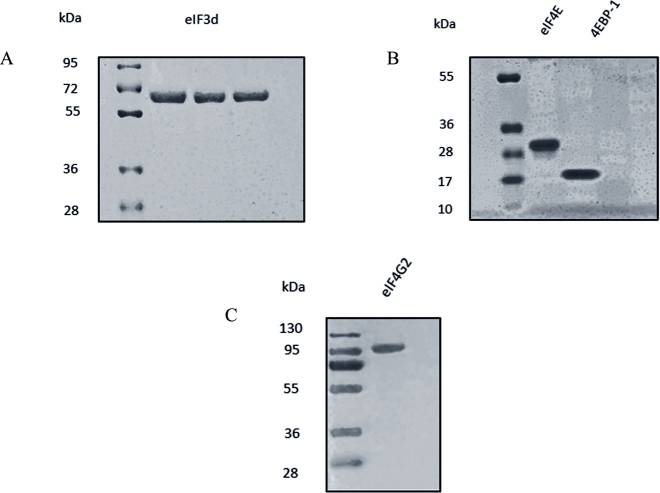


**Revised supplementary Figure 3:**
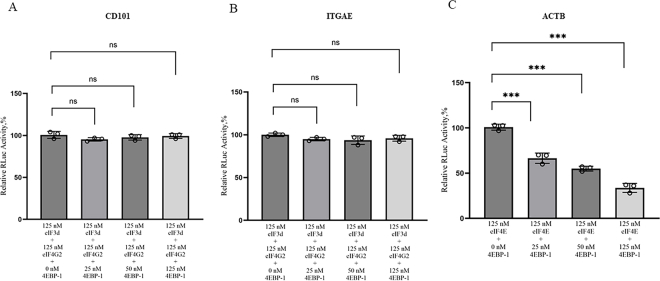


## Supporting information

Raw data sheet

Supplementary table

Supplementary fig captions

## References

[1] Jackson R.J., Hellen C.U., Pestova T.V. (2010). The mechanism of eukaryotic translation initiation and principles of its regulation. Nat. Rev. Mol. Cell Biol..

[2] Sonenberg N., Hinnebusch A.G. (2009). Regulation of translation initiation in eukaryotes: mechanisms and biological targets. Cell.

[3] Hinnebusch A.G., Lorsch J.R. (2012). The mechanism of eukaryotic translation initiation: new insights and challenges. Cold Spring Harb. Perspect. Biol..

[4] Dever T.E., Kinzy T.G., Pavitt G.D. (2016). Mechanism and regulation of protein synthesis in Saccharomyces cerevisiae. Genetics.

[5] Brito Querido J., Díaz-López I., Ramakrishnan V. (2024). The molecular basis of translation initiation and its regulation in eukaryotes. Nat. Rev. Mol. Cell Biol..

[6] Batool A., Aashaq S., Andrabi K.I. (2019). Eukaryotic initiation factor 4E (eIF4E): a recap of the cap-binding protein. J. Cell Biochem..

[7] Aitken C.E., Lorsch J.R. (2012). A mechanistic overview of translation initiation in eukaryotes. Nat. Struct. Mol. Biol..

[8] Merrick W.C., Pavitt G.D. (2018). Protein synthesis initiation in eukaryotic cells. Cold Spring Harb. Perspect. Biol..

[9] Robichaud N., Sonenberg N., Ruggero D., Schneider R.J. (2019). Translational control in cancer. Cold Spring Harb. Perspect. Biol..

[10] Petrychenko V., Yi S.H., Liedtke D., Peng B.Z., Rodnina M.V., Fischer N. (2025). Structural basis for translational control by the human 48S initiation complex. Nat. Struct. Mol. Biol..

[11] Holcik M., Sonenberg N. (2005). Translational control in stress and apoptosis. Nat. Rev. Mol. Cell Biol..

[12] Williams T.D., Rousseau A. (2024). Translation regulation in response to stress. FEBS J.

[13] Magagnin M.G., van den Beucken T., Sergeant K., Lambin P., Koritzinsky M., Devreese B. (2008). The mTOR target 4E-BP1 contributes to differential protein expression during normoxia and hypoxia through changes in mRNA translation efficiency. Proteomics.

[14] Gingras A.C., Gygi S.P., Raught B., Polakiewicz R.D., Abraham R.T., Hoekstra M.F. (1999). Regulation of 4E-BP1 phosphorylation: a novel two-step mechanism. Genes Dev..

[15] Qin X., Jiang B., Zhang Y. (2016). 4E-BP1, a multifactor regulated multifunctional protein. Cell Cycle.

[16] Böhm R., Imseng S., Jakob R.P., Hall M.N., Maier T., Hiller S. (2021). The dynamic mechanism of 4E-BP1 recognition and phosphorylation by mTORC1. Mol. Cell.

[17] Alard A., Katsara O., Rios-Fuller T., Parra C., Ozerdem U., Ernlund A. (2023). Breast cancer cell mesenchymal transition and metastasis directed by DAP5/eIF3d-mediated selective mRNA translation. Cell Rep..

[18] Volta V., Pérez-Baos S., de la Parra C., Katsara O., Ernlund A., Dornbaum S. (2021). A DAP5/eIF3d alternate mRNA translation mechanism promotes differentiation and immune suppression by human regulatory T cells. Nat. Commun..

[19] Evdokimova V., Tognon C.E., Sorensen P.H. (2012). On translational regulation and EMT. Semin. Cancer Biol..

[20] Mahé M., Rios-Fuller T., Katsara O., Schneider R.J. (2024). Non-canonical mRNA translation initiation in cell stress and cancer. NAR Cancer.

[21] Lee A.S., Kranzusch P.J., Cate J.H. (2015). eIF3 targets cell-proliferation messenger RNAs for translational activation or repression. Nature.

[22] Lee A.S., Kranzusch P.J., Doudna J.A., Cate J.H. (2016). eIF3d is an mRNA cap-binding protein that is required for specialized translation initiation. Nature.

[23] Thompson L., Depledge D.P., Burgess H.M., Mohr I. (2022). An eIF3d-dependent switch regulates HCMV replication by remodeling the infected cell translation landscape to mimic chronic ER stress. Cell Rep..

[24] Lamper A.M., Fleming R.H., Ladd K.M., Lee A.S.Y. (2020). A phosphorylation-regulated eIF3d translation switch mediates cellular adaptation to metabolic stress. Science.

[25] Ma S., Liu J.Y., Zhang J.T. (2023). eIF3d: A driver of noncanonical cap-dependent translation of specific mRNAs and a trigger of biological/pathological processes. J. Biol. Chem..

[26] Asano K., Vornlocher H.P., Richter-Cook N.J., Merrick W.C., Hinnebusch A.G., Hershey J.W. (1997). Structure of cDNAs encoding human eukaryotic initiation factor 3 subunits. Possible roles in RNA binding and macromolecular assembly. J. Biol. Chem..

[27] Zhou Y., Chai R., Wang Y., Yu X. (2024). Deciphering eIF3d's role in immune regulation and malignant progression: a pan-cancer analysis with a focus on colon adenocarcinoma. J. Inflamm. Res..

[28] Lin Y., Zhang R., Zhang P. (2016). Eukaryotic translation initiation factor 3 subunit D overexpression is associated with the occurrence and development of ovarian cancer. FEBS Open Bio..

[29] He J., Wang X., Cai J., Wang W., Qin X. (2017). High expression of eIF3d is associated with poor prognosis in patients with gastric cancer. Cancer Manag Res..

[30] Zhang F., Xiang S., Cao Y., Li M., Ma Q., Liang H. (2017). eIF3d promotes gallbladder cancer development by stabilizing GRK2 kinase and activating PI3K-AKT signaling pathway. Cell Death Dis..

[31] Wang D., Jia Y., Zheng W., Li C., Cui W. (2019). Overexpression of eIF3d in lung adenocarcinoma is a new independent prognostic marker of poor survival. Dis. Markers.

[32] Huang H., Gao Y., Liu A., Yang X., Huang F., Xu L. (2019). eIF3d promotes sunitinib resistance of renal cell carcinoma by interacting with GRP78 and inhibiting its degradation. EBioMedicine.

[33] Shestakova E.D., Smirnova V.V., Shatsky I.N., Terenin I.M. (2023). Specific mechanisms of translation initiation in higher eukaryotes: the eIF4G2 story. RNA.

[34] Weingarten-Gabbay S., Khan D., Liberman N., Yoffe Y., Bialik S., Das S. (2014). The translation initiation factor DAP5 promotes IRES-driven translation of p53 mRNA. Oncogene.

[35] Yamanaka S., Zhang X.Y., Maeda M., Miura K., Wang S., Farese R.V. (2000). Essential role of NAT1/p97/DAP5 in embryonic differentiation and the retinoic acid pathway. EMBO J.

[36] Virgili G., Frank F., Feoktistova K., Sawicki M., Sonenberg N., Fraser C.S. (2013). Structural analysis of the DAP5 MIF4G domain and its interaction with eIF4A. Structure.

[37] Lee S.H., McCormick F. (2006). p97/DAP5 is a ribosome-associated factor that facilitates protein synthesis and cell proliferation by modulating the synthesis of cell cycle proteins. EMBO J.

[38] Haizel S.A., Bhardwaj U., Gonzalez R.L., Mitra S., Goss D.J. (2020). 5'-UTR recruitment of the translation initiation factor eIF4GI or DAP5 drives cap-independent translation of a subset of human mRNAs. J. Biol. Chem..

[39] Whittaker A., Goss D.J. (2024). Modeling the structure and DAP5-binding site of the FGF-9 5'-UTR RNA utilized in cap-independent translation. RNA.

[40] Marash L., Liberman N., Henis-Korenblit S., Sivan G., Reem E., Elroy-Stein O. (2008). DAP5 promotes cap-independent translation of Bcl-2 and CDK1 to facilitate cell survival during mitosis. Mol. Cell.

[41] de la Parra C., Ernlund A., Alard A., Ruggles K., Ueberheide B., Schneider R.J. (2018). A widespread alternate form of cap-dependent mRNA translation initiation. Nat. Commun..

[42] Wypijewska A., Bojarska E., Lukaszewicz M., Stepinski J., Jemielity J., Davis R.E. (2012). 7-methylguanosine diphosphate m(7)GDP is not hydrolyzed but strongly bound by decapping scavenger (DcpS) enzymes and potently inhibits their activity. Biochemistry.

[43] Walczak S., Nowicka A., Kubacka D., Fac K., Wanat P., Mroczek S. (2017). A novel route for preparing 5' cap mimics and capped RNAs: phosphate-modified cap analogues obtained via click chemistry. Chem. Sci..

[44] Roiuk M., Neff M., Teleman A.A. (2024). eIF4E-independent translation is largely eIF3d-dependent. Nat. Commun..

[45] Shin S., Han M.J., Jedrychowski M.P., Zhang Z., Shokat K.M., Plas D.R. (2023). mTOR inhibition reprograms cellular proteostasis by regulating eIF3d-mediated selective mRNA translation and promotes cell phenotype switching. Cell Rep..

[46] Szostak E., García-Beyaert M., Guitart T., Graindorge A., Coll O., Gebauer F. (2018). Hrp48 and eIF3d contribute to msl-2 mRNA translational repression. Nucleic Acids Res..

[47] Hayek H., Gross L., Janvier A., Schaeffer L., Martin F., Eriani G. (2021). eIF3 interacts with histone H4 messenger RNA to regulate its translation. J. Biol. Chem..

[48] Masutani M., Sonenberg N., Yokoyama S., Imataka H. (2007). Reconstitution reveals the functional core of mammalian eIF3. EMBO J.

[49] Smith M.D., Arake-Tacca L., Nitido A., Montabana E., Park A., Cate J.H. (2016). Assembly of eIF3 mediated by mutually dependent subunit insertion. Structure.

[50] Wagner S., Herrmannová A., Šikrová D., Valášek L.S. (2016). Human eIF3b and eIF3a serve as the nucleation core for the assembly of eIF3 into two interconnected modules: the yeast-like core and the octamer. Nucleic Acids Res..

[51] Saha B., Bhardwaj U., Goss D.J. (2023). Thermodynamically favorable interactions between eIF4E binding domain of eIF4GI with structured 5'-untranslated regions drive cap-independent translation of selected mRNAs. Biochemistry.

