# Supplementary material for: eIF3d and eIF4G2 mediate an alternative mechanism of cap-dependent but eIF4E-independent translation initiation
Source: J Biol Chem. 2025 Feb 17;301(4):108317. doi: 10.1016/j.jbc.2025.108317 (PMC11968281; doi:10.1016/j.jbc.2025.108317)
Supplement: Supplementary table [file mmc2.docx]

eIF3d and eIF4G2 mediate an alternative form of cap-dependent but eIF4E-independent mechanism of human mRNA translation initiation

Jacob N. K. Quartey^a, b^ and Dixie J. Goss^a,b,c*^

*^a^Ph.D. Program in Biochemistry, The Graduate Center of the City University of New York, New York, NY*

*10016;*

*^b^Department of Chemistry, Hunter College* *of the City University of New York, New York, NY 10065;*

*^c^Ph.D. Program in Chemistry, The Graduate Center of the City University of New York, New York, NY 10016*

*** Corresponding author: Dixie J. Goss

Email: [dgoss@hunter.cuny.edu](mailto:dgoss@hunter.cuny.edu)

Table S1. 5’UTR sequences of mRNA transcripts

| 5’UTR | Number of nucleotides | Sequence |
| --- | --- | --- |
| CD101 | 66 | UUAAGGUCACUCAACCUCUGAAUGUUAGUGACACUAUUGGGACGAAAAAGGACUGUGCUGGCCGAU |
| ITGAE | 99 | CCGCCUCCUGGCCUCCUGGCUGAGGGGAAGCUGAGUGGGCCACGGCCCAUGUGUCGCACUCGCCUCGGCUCCCACACAGCCGCCUCUGCU CCAGCAAGG |
| ACTB | 84 | ACCGCCGAGACCGCGUCCGCCCCGCGAGCACAGAGCCUCGCCUUUGCCGAUCCGCCGCCCGUCCACACCCGCCGCCAGCUCACC |
