# Supplementary material for: eIF3d and eIF4G2 mediate an alternative mechanism of cap-dependent but eIF4E-independent translation initiation
Source: J Biol Chem. 2025 Feb 17;301(4):108317. doi: 10.1016/j.jbc.2025.108317 (PMC11968281; doi:10.1016/j.jbc.2025.108317)
Supplement: Supplementary fig captions [file mmc3.docx]

**Supplementary Information**

**Figure Captions**

Figure S1. **SDS-PAGE gel diagrams showing purified protein products.** (*A*) eIF3d, (*B*) eIF4E and 4EBP-1, and (*C*) eIF4G2.

Figure S2. **Differential effects of increasing concentrations of eIF3d/eIF4G2 and eIF4E on the translation of ACTB mRNA.** Bar heights and error bars correspond to the average and standard deviations, respectively, of three independent luciferase activity measurements. Data was analyzed by two-tailed unpaired Student’s *t*-test; ***, p < 0.001.

Figure S3. **4EBP-1 effects on eIF3d/eIF4G2 and eIF4E mediated cap-dependent translation.** Differential effects of 4EBP-1 on eIF3d/eIF4G2 mediated cap-dependent translation (*A*-*B*) and eIF4E mediated cap-dependent translation (*C*). Bar heights and error bars correspond to the average and standard deviations, respectively, of three independent luciferase activity measurements. Data was analyzed by two-tailed unpaired Student’s *t*-test; ns, p = 0.12, ***, p < 0.001.
